# Gray Matter Deficits and Dysfunction in the Insula Among Individuals With Intermittent Explosive Disorder

**DOI:** 10.3389/fpsyt.2020.00439

**Published:** 2020-05-20

**Authors:** Ji-Woo Seok, Chaejoon Cheong

**Affiliations:** ^1^ Department of Psychiatry, University of Nebraska Medical Center, Omaha, NE, United States; ^2^ Department of Rehabilitation Counseling Psychology, Seoul Hanyoung University, Seoul, South Korea; ^3^ Bioimaging Research Team, Korean Basic Science Institute, Cheongju, South Korea

**Keywords:** 7T magnetic resonance imaging, functional magnetic resonance imaging, insula, intermittent explosive disorder, voxel-based morphometry

## Abstract

Although numerous neuroimaging studies have evaluated the characteristics of intermittent explosive disorder (IED), studies on the structural alterations and focal dysfunction in the brain in this condition are limited. This study aimed to identify gray matter deficits and functional alterations in individuals with IED using voxel-based morphometry (VBM) and functional magnetic resonance imaging (fMRI) analyses. Fifteen men with IED and 15 age- and sex-matched healthy controls participated in this study. Gray matter volume and brain activation while viewing the anger-inducing films were measured using 7T MRI. VBM results indicated that individuals with IED had significantly reduced gray matter volume in the insula, amygdala, and orbitofrontal area, relative to controls. Gray matter volume in the left insula was negatively correlated with composite aggression scores. fMRI results demonstrated that relative to healthy controls, individuals with IED showed greater activation in the insula, putamen, anterior cingulate cortex, and amygdala during anger processing. Left insular activity was positively correlated with composite aggression scores. Collectively, these findings suggest that structural and functional alterations in the left insula are linked to IED; this provides insight into the neural mechanisms underlying IED.

## Introduction

Self-protective aggression lies within the normal range of human behavior. However, impulsive and intentional aggression behaviors are viewed as pathological. Impulsive and intentional aggression behaviors are known to have significant physical and psychosocial effects on the aggressive individual, victims of the aggression, and society (1). Pathological impulsive aggression, despite its serious negative consequences, is defined as intermittent explosive disorder (IED) in the Diagnostic and Statistical Manual of Mental Disorders, Fifth Edition (DSM-5) (2). Previous research demonstrates that the lifetime prevalence of IED is 3%–5% (1) and that the average age of onset is 14 years (3). Studies on IED identified that individuals with IED display 65–70 acts of assault and property destruction on an average, and this frequency of aggressive acts is much higher than that among individuals without IED (3, 4). These studies also demonstrated that IEDs incur large costs including over $1,000 in damage, and lead to multiple hospitalizations (3, 4). Owing to the adverse personal and social problems arising from IED, research is needed into the behavioral and neurobiological underpinnings of IED to identify the theoretical and clinical implications of this disorder, and to discover effective treatments.

Neuroimaging studies demonstrate that IED is related to possible brain impairments (5–11). In a functional magnetic resonance imaging (fMRI) study on face processing, individuals with IED showed a stronger amygdala response to angry faces than did healthy controls (6, 8, 10), and amygdala activation to angry faces was correlated with a lifetime history of aggression (6) and the number of prior aggressive acts (10). Compared with controls, individuals with IED exhibit diminished functional connectivity between the amygdala and orbitofrontal cortex, suggesting reduced regulatory control of the prefrontal cortex (10). Other fMRI studies on regulation ability among patients with IED demonstrated that these individuals displayed more errors on the color-word Stroop task than did controls, and revealed that this inhibitory dysregulation was related to impairments in the dorsolateral prefrontal cortex (11).

Structural brain imaging has been used extensively in recent years to examine the morphology of brain tissues. In particular, structural brain imaging of individuals with mental disorders provides information regarding cortical atrophy, thinning, and deformation that occurs in response to psychiatric disorders. Mounting evidence from structural brain imaging studies has revealed that IED may be linked to possible anatomical changes within the brain (5, 9). Lee et al. (9) suggested that IED is associated with the degeneration of white matter tracts in long-range connections between frontal and temporoparietal regions. In addition, another structural study using voxel-based morphometry (VBM) revealed significantly reduced gray matter volume in patients with IED relative to controls in frontolimbic systems including the orbitofrontal cortex, ventral medial prefrontal cortex, anterior cingulate cortex, amygdala, insula, and uncus (5).

VBM analysis is used to investigate the focal differences in brain anatomy *in vivo* using structural MRI. This type of analysis is useful for detecting small-scale regional alterations across the volume of the whole brain or its subparts in individuals with neurological and psychiatric disorders (12). However, as VBM depends entirely on accurate registration and normalization, it is likely to increase false estimates (13, 14). Diffeomorphic Anatomical Registration Through Exponentiated Lie Algebra (DARTEL) algorithms have been proposed to reduce false estimates by improving intersubject registration of brain images (15). DARTEL can consequently strengthen the robustness and reliability of VBM results (16, 17).

Although several neuroimaging studies have reported impaired brain function and structure in IED, few studies have used both, structural and functional analyses to focus on the functional and structural alterations in IED. Integrating measures of structural and functional brain imaging provides profound insights into the neurobiological underpinnings of IED and may lead to the detection of clinically useful biomarkers for IED. Therefore, in this study, we used 7T MRI to identify alterations in brain structure and function among individuals with IED, by measuring gray matter volume and brain function during anger processing from combined fMRI and automated whole-brain VBM DARTEL analyses. In particular, we focused on gray matter volume in the frontolimbic system, including the amygdala, prefrontal cortex, and insula, and investigated alterations in brain function in areas exhibiting reduced gray matter volume.

## Materials and Methods

### Participants

Fifteen right-handed men diagnosed with IED and 15 right-handed healthy controls with no history of any psychiatric or personality disorder participated in the study. All participants were 25–35 years old and were recruited from local psychiatric clinics and counseling centers, as well as through website postings. All participants in the IED group met DSM-5 criteria for current IED. Additional diagnoses were also used based on the Structured Clinical Interview for DSM-IV Axis I Disorders (SCID-I) and Symptom Check List-90-Revised (SCL-90-R). To identify the specific characteristics for IED, the IED participants were excluded from the study if they reported any of the following: (1) on current psychopharmacological medication, (2) a history of bipolar or psychotic disorder, (3) a traumatic head injury, or (4) a current major depressive episode or substance dependence.

For the control group, 15 participants with matching demographic characteristics (age, gender, education level, and income level) were selected and none of the 15 control participants fulfilled the criteria for any psychiatric disorder. None of the participants had any history of brain injury or abuse of psychoactive drugs within the past year. The patients with IED were not on any medication related to IED at the time of the study. All participants provided written informed consent according to the Declaration of Helsinki (18) after being thoroughly informed about the details of the experiment. The experimental procedures were approved by the Chungnam National University Institutional Review Board (approval number: 201803-SB-041-01).

### Psychological Assessments

To assess the severity of aggression, a composite aggression score was obtained by measuring the three aggression scales including the Life History of Aggression (19), Buss-Perry Aggression Questionnaire (20), and State-Trait Anger Expression Inventory-2 (21) in all participants. The Life History of Aggression assesses a lifetime history of actual aggressive behavior, and the Buss-Perry Aggression Questionnaire assesses aggressive tendency by measuring the four components of physical aggression, verbal aggression, anger, and hostility. The State-Trait Anger Expression Inventory-2 assessment evaluates the temporary condition of anger experience (state anger) and the more long-standing propensity of anger experience (trait anger).

As there are strong correlations between the three aggression scales (*r* > 0.8, *p* < 0.01), we created composite variables for aggression in a data-reduction step summing the z-scores on these three scales (22). The Beck Depression Inventory (23), Beck Anxiety Inventory (24), and Barratt Impulsiveness Scale II (25) were administered to identify other psychological characteristics among all the participants. Statistical analyses were conducted with SPSS 25 (SPSS Inc., Chicago, IL, USA). The independent t test and Mann-Whitney U test were used for between-group comparisons of demographic and clinical variables. Data on the demographic and clinical characteristics of all participants have been presented in **Table 1** and **Table S1**.

**Table 1:** Demographic and clinical characteristics of the IED and control groups.

Variables (mean ± SD)	IED	HC	p	Effect Size
**Age (year)**	28.53 ± 2.36	28.60 ± 4.40	0.95	0.02
**Onset age of FAA**	13.93 ± 1.79	N.A.	N.A	N.A.
**Cigarettes per day** **(Last 3 months)**	5.53 ± 2.56	4.53 ± 2.85	0.32	0.37
Num. of alcohol use day* (Last 3 months)	2.00 [1.00, 3.00]	2.00 [1.00, 3.00]	0.91	0.001
Num. of AO per week* **(Last 3 month)**	4.00 [3.00, 5.00]	0.00 [0.00, 0.50]	0.00	0.72
BDI*	9.00 [3.50, 16.50]	2.00 [0.50, 9.00]	0.04	0.14
BAI*	8.00 [4.00, 12.00]	3.00 [1.00, 4.50]	0.09	0.09
**BIS-II**	67.00 ± 6.77	60.07 ± 8.35	0.02	0.91
**LHA**	11. 67 ± 3.64	4.73 ± 3.41	0.00	1.95
**SA**	21.93 ± 4.13	15.13 ± 2.90	0.01	0.95
**TA**	23.00 ± 4.58	16.00 ± 3.95	0.00	1.63
**AQ**	70.07 ± 5.34	47.93 ± 15.31	0.00	1.93

AO, aggressive outbursts; AQ, aggression questionnaire; BAI, Beck Anxiety Inventory; BDI, Beck Depression Inventory; BIS, Barrett's Impulsiveness Scale II; FAA, First Anger Attack HC, healthy controls; IED, intermittent explosive disorder; LHA, Life History of Aggression Questionnaire; Num, Number; SA, state anger; TA, trait anger.

* The variables are nonnormal distributed and are described as median and interquartile range. The Mann-Whitney U test was used to compare between groups for the variables.

### Stimuli and Experimental Paradigm

**Figure 1:**
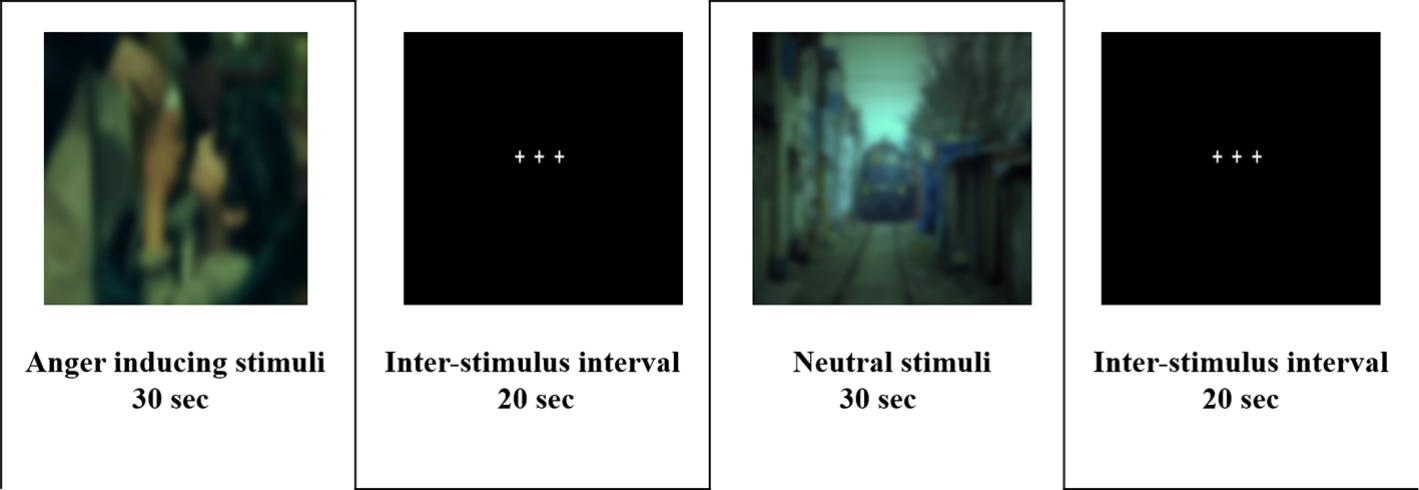
Experimental paradigm for functional magnetic resonance imaging.

Two types of film clips (anger and neutral) were presented to the participants. Each film clip was a scene selected from movies, dramas, news, and/or documentaries. Anger-inducing clips included stories of child abuse, mocking a person with disabilities, racism, unfair treatment, and bullying. Neutral clips showed nonaffective nature scenes and were similar in color and hue to those of anger-inducing clips. Seok and Cheong (26) have previously employed these stimuli (**Figure 1**) (26).

The fMRI task was divided into 10 blocks, with five neutral and five anger-inducing blocks. Each block included a film clip and an interstimulus interval, and each film clip presented for 30 s in random order to eliminate order effects. A 12-s interstimulus interval was presented between blocks to reduce possible carry over effects. The duration of the entire scanning session was approximately 7 min (**Figure 1**).

After the scanning procedure, participants were asked the following question to assess suitability, frequency and intensity of anger during viewing the film clips; “Does this film clip provoke the anger? Please answer yes or no,” “How often did you get angry during viewing this film clip? Please answer the number,” “How angry you were while watching this film clip? Please rate the intensity on below scale ranging from 0 (not angry at all) to 5 (extremely angry).”

### Data Acquisition

A 7T Philips Achieva MRI scanner (Philips Medical Systems, Best, Netherlands) was used for image acquisition. High-resolution structural images were obtained using a three-dimensional T1-weighted sequence (repetition time = 5.5 ms, echo time = 2.6 ms, flip angle = 7, field of view = 234 × 234 mm^2^, voxel size = 0.7 × 0.7 × 0.7 mm^3^). During the fMRI experiment, blood oxygenation level dependent images were acquired using a T2*-weighted gradient echo-planar imaging sequence with the following parameters: repetition time = 2000 ms, echo time = 17 ms, flip angle = 70°, bandwidth = 1856 Hz/pixel, field of view = 192 × 192 mm^2^, voxel size = 2.0 × 2.0 × 3.5 mm^3^, and 32 interleaved slices with no slice gap.

### VBM Analysis

VBM analysis was conducted using SPM12 (http://www.fil.ion.ucl.ac.uk/spm) implemented in Matlab R2017a (MathWorks, Natick, MA). First, T1-weighted images were segmented into gray and white matter using the standard unified segmentation model. Second, gray matter templates were made from the entire image dataset using the diffeomorphic nonlinear registration algorithm (DARTEL) technique to increase the accuracy of intersubject registration by estimating the nonlinear deformation method (13, 15). Third, each participant's gray matter image was normalized to Montreal Neurological Institute space (http://www.mni.mcgill.ca/) using an initial affine registration and nonlinear warping. Fourth, modulation was performed to ensure that relative volumes of gray matter were preserved after the spatial normalization procedure. Lastly, images were smoothed with an 8-mm, full-width-at-half-maximum Gaussian kernel to decrease spatial noise.

After data preprocessing, between-group comparisons were conducted using analysis of covariance (ANCOVA), with age, education, BDI score, BAI score, BIS scores, and intracranial volume (i.e., the sum of gray matter, white matter, and cerebrospinal fluid, as derived from the segmentation process) as nuisance variables. To estimate the relationship between the severity of aggression and structural abnormalities linked to depression, anxiety, and impulsivity, whole-brain analyses using multiple linear regression were performed between the gray matter volume and the composite aggression score. The exploratory lenient significance threshold was set at *p* < 0.001 (uncorrected at the voxel level) and *p* < 0.05 (corrected for spatial extent).

To confirm the validity of the results acquired using the whole-brain analysis, *post hoc* analysis was conducted with the extracted gray matter volume from each brain area using SPSS 25.

### Functional MRI Analysis

We used SPM12 (http://www.fil.ion.ucl.ac.uk/spm) implemented in Matlab R2017a (MathWorks) for preprocessing and statistical analyses. The first three image volumes were discarded to prevent instability of the initial MRI signal. Correction of slice acquisition timing, realignment to the first functional image using an affine (six parameter) spatial transformation, correction of geometric distortion by the unwarp function, coregistration with the high-resolution anatomical image, and normalization to the standard brain of the Montreal Neurological Institute were executed after the origin coordinates were adjusted to the anterior commissure. Lastly, smoothing was performed with a 6-mm full-width-at-half-maximum Gaussian kernel.

A design matrix was constructed for the anger-evoking and neutral conditions using a box-car function convolved with the canonical hemodynamic response function and its temporal derivative. The six movement parameters of rigid body transformation applied by the realignment procedure were included as nuisance variables in the model. A high-pass filter was implemented using a discrete cosine transform set with a cutoff frequency of 1/128 Hz in the design matrix. Since 7T MRI data have several limitations including signal dropout, distortions, and susceptibility, we used the following methods to compensate for them: (1) applying second-order B0 shimming, (2) using barium titanate–based dielectric pads (27), and (3) eliminating data with over 20% signal dropout in the ventromedial prefrontal cortex.

T-contrasts were calculated for each participant to identify anger-specific neural substrates by comparing the anger-inducing and neutral conditions. To measure group-level activation of the blood oxygenation level-dependent response during anger processing, the contrast images of all participants in each group were subjected to a standard random-effects analysis. ANCOVA analysis was applied to control for potential confounding variables using BDI, BAI, and BIS scores as covariates. The threshold for statistical significance was set at *p* < 0.001 or *p* < 0.05 with false discovery rate (FDR) correction. Additionally, multiple linear regression was conducted to determine the regions of activation that correlated with the severity of aggression (i.e., composite aggression score) after adjusting for depression (i.e., BDI score), anxiety (i.e., BAI score), and impulsivity (i.e., BIS score) effects.

To confirm the validity of the results acquired using ANCOVA and multiple linear regression analyses, *post hoc* analysis was conducted with the percent signal change from regions of interest (ROIs) using SPSS 25. The percent signal change was extracted from the ROIs based on the results of the between-group and correlation analyses (i.e., bilateral putamen, bilateral insula, right amygdala, right anterior cingulate cortex). The ROIs were created by placing a 5-mm sphere around the described coordinates in **Tables 2** and **3**.

**Table 2:** The result of anger induction.

Variables (mean ± SD)	IED	HC	p
**Frequency of anger induction (%)** ^**a**^	40.00 [40.00, 50.00]	40.00 [30.00, 50.00]	0.43
**Intensity of anger** ^**b**^	18.53 ± 3.31	14.67 ± 5.40	0.03

HC, healthy controls; IED, intermittent explosive disorder.

a Represented as percentage of anger inducing stimuli that evoked anger among five anger-inducing clips. The variable is nonnormal distributed and is described as median and interquartile range. The Mann-Whitney U test was used to compare between groups for the variable.

b Degree of anger triggered by the anger-inducing cues on a five-point Likertscale.

**Table 3:** Differences in gray matter volume between the IED and control groups and brain regions negatively correlated with the severity of aggression.

Brain region	MNI coordinates			*t*_max_	Cluster size **(voxels)**
	x	y	z		
IED < HC^1^					
Left OFC	−15	60	0	4.04	171
Left insula (BA 13) Right insula (BA 13)	−28 33	27 30	0 −5	4.29 3.91	204 102
Left amygdala	−21	-6	−9	3.55	39
Correlation with the severity of aggression^2^					
Left insula	−24	33	−2	3.61	43

BA, Brodmann area; HC, healthy control; IED, intermittent explosive disorder; MNI, Montreal Neurological Institute; OFC, orbitofrontal cortex.

MNI coordinates of the maximum t-scores are shown for each cluster.

1 Results are reported at p < 0.001, uncorrected for the whole-brain analysis. The regions exhibited the greater activity in the IED group compared to healthy controls during the anger-inducing condition compared to the neutral condition. There was no brain region in which healthy controls exhibited greater activation than the IED group.

2 Results are reported at p < 0.001, uncorrected for the whole-brain analysis.

## Results

### Participant Characteristics and Psychological Assessments

Healthy controls and individuals with IED did not differ significantly in age (*t* = 0.05, *p* > 0.05, Cohen's *d* = 0.02) or Beck Anxiety Inventory score (Mann-Whitney U =153, *p* > 0.05, η^2^ = 0.09). Individuals with IED scored higher on the Barratt Impulsiveness Scale II (*t* = 2.50, *p* < 0.05, Cohen's *d* = 0.91), Beck Depression Inventory (Mann-Whitney U =162.5, *p* < 0.05, η^2^ = 0.14), Life History of Aggression (*t* = 5.34, *p* < 0.001, Cohen's *d* = 1. 95), state anger (*t* = 2.61, *p* < 0.05, Cohen's *d* = 0.95), trait anger (*t* = 4.48, *p* < 0.001, Cohen's *d* = 1.63), and Buss-Perry Aggression Questionnaire (*t* = 5.29, *p* < 0.001, Cohen's *d* = 1.93) than healthy controls (**Table 1**).

### Anger Induction

All participants reported that they had been angry during the anger-inducing conditions and had not felt any emotion while viewing the neutral pictures. The result of the Mann-Whitney U test did not show group differences in the frequency of occurrence of angry feelings when viewing the five anger-inducing stimuli (Mann-Whitney U = 131, p > 0.05, η^2^ = 0.28). However, Independent t-tests of the intensity of angry feelings indicated that compared to healthy controls, individuals with IED exhibited anger that was more intense in response to anger-inducing stimuli (t = 2.29, p < 0.05, Cohen's d = 0.71) (**Table 2**).

### VBM Analysis

**Figure 2:**
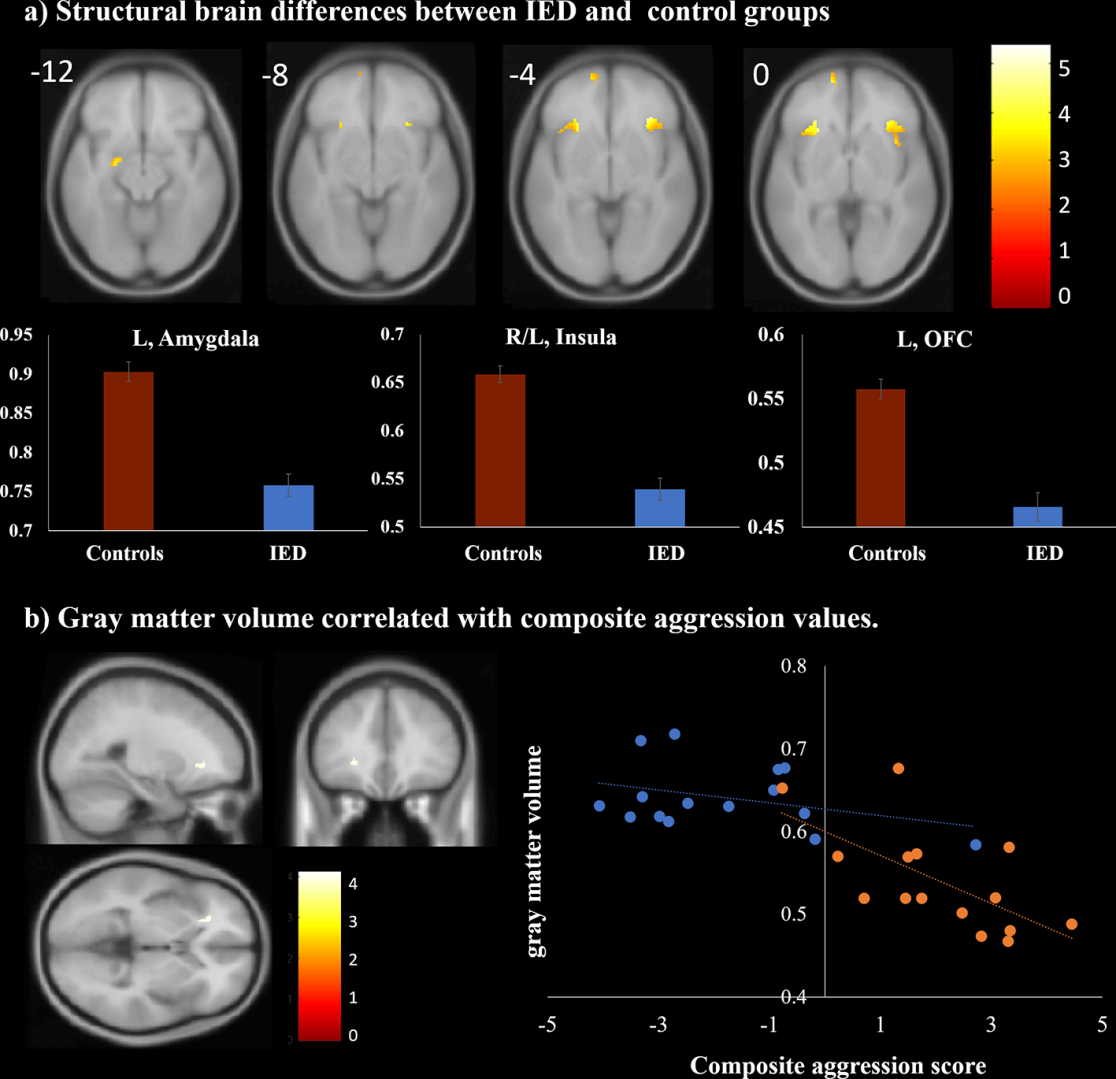
Structural magnetic resonance imaging findings of individuals with intermittent explosive disorder (IED) and healthy controls. **(A)** Regions showing gray matter reduction in individuals with IED relative to controls [p < 0.05, false discovery rate (FDR) corrected]. **(B)** Brain region map and scatter plots of the correlation between the composite aggression score and the gray matter volume in the left insula (p < 0.001, uncorrected). Each orange and blue circle represents the data of an IED and control participant, respectively.

VBM analysis was conducted to detect structural differences between individuals with IED and healthy controls. As shown in **Table 3** and **Figure 2A**, individuals with IED had significantly reduced gray matter volume in the left amygdala, left orbitofrontal cortex, and left anterior insula when compared with healthy controls (*p* < 0.001, uncorrected). No areas were identified where healthy controls showed reduced gray matter volume relative to that of the IED group. A significant negative correlation was detected between the gray matter volume in the left insula and the severity of aggression (i.e., composite aggression scores) as shown in **Figure 2B** (*p* < 0.001, uncorrected). The results of *post hoc* analyses using multiple linear regression revealed that the gray matter volume in the left insula was negatively linked to the composite aggression score in IED group.

### fMRI Analysis

**Figure 3:**
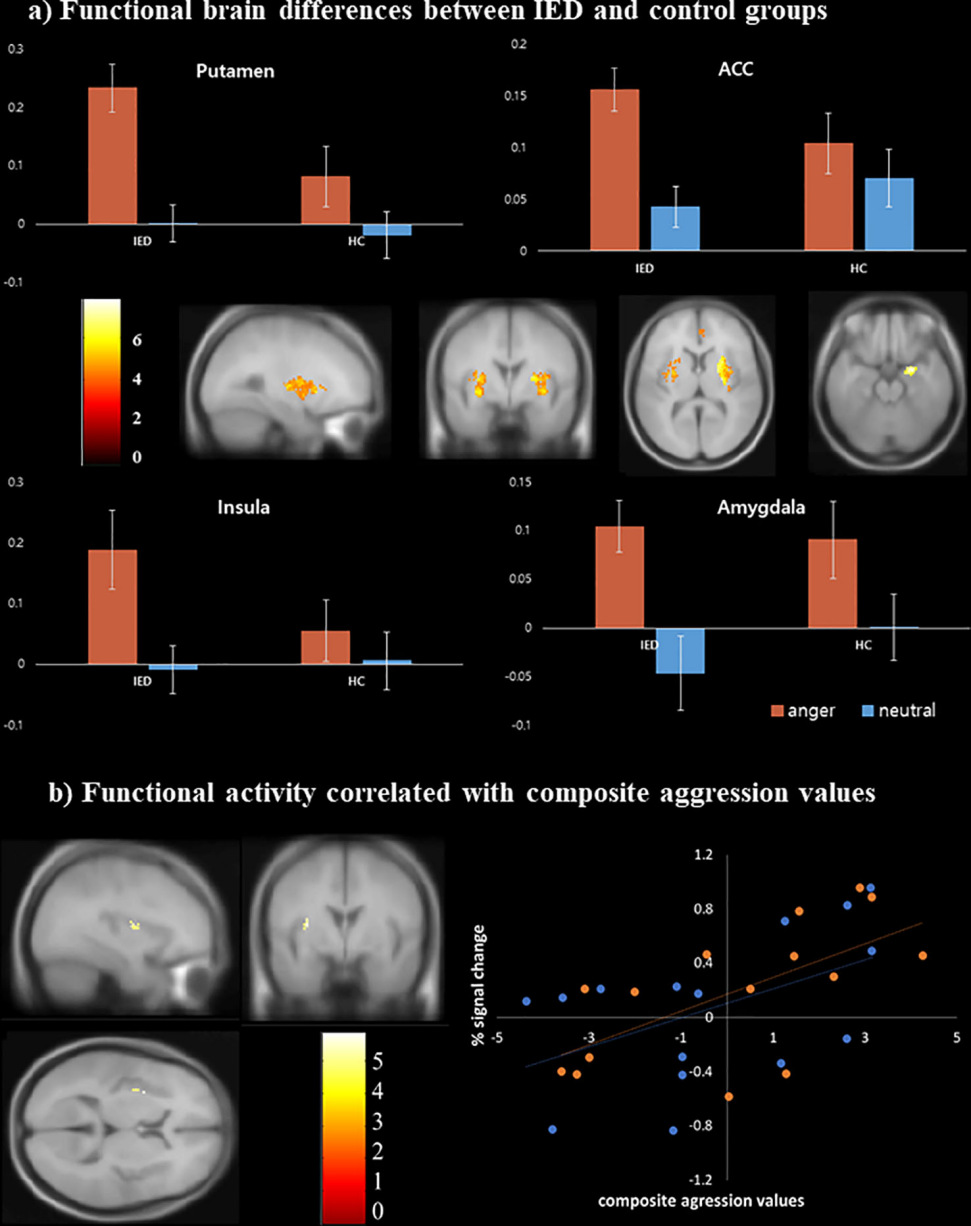
Functional magnetic resonance imaging findings of individuals with intermittent explosive disorder (IED) and healthy controls. **(A)** Brain areas showing increased activity in response to anger stimuli among individuals with IED compared to healthy controls [p < 0.05, false discovery rate (FDR) corrected]. **(B)** Scatter plots of the correlation between the composite aggression score and functional activity in the left insula (p < 0.001, uncorrected). Each orange and blue circle represents the data of an IED and control participant, respectively.

In both groups, activation was observed in the bilateral middle/inferior frontal gyri (Brodmann area [BA] 9), cuneus/precuneus (BA 7, 18, 19), thalamus, cingulate gyri (BA 24, 32), anterior insula (BA 13), putamen, amygdala, and superior/middle temporal gyri in response to the anger-inducing vs. neutral condition (*p* < 0.05, FDR corrected). Between-group analyses revealed that compared to healthy controls, individuals with IED showed significantly more activation in the bilateral putamen, bilateral anterior insula (BA 13), right amygdala, and right anterior cingulate cortex (BA 24) during the anger-inducing vs. neutral condition. There was no brain region in which healthy controls exhibited greater activation than the IED group (**Table 4**, **Figure 3A**) (*p* < 0.05, FDR corrected). **Figure 3A** illustrates the percent signal changes in the selected ROIs based on the results of the between-group analyses in healthy controls and individuals with IED for each experimental condition (i.e., anger-inducing and neutral conditions).

**Table 4:** Functional magnetic resonance imaging (fMRI) results from analysis of covariance.

Brain region	MNI coordinates			*t*_max_	Cluster size (voxels)
	x	y	z		
IED > HC					
Left putamen Right putamen	−26 24	8 10	8 6	6.70 8.47	650 809
Left insula (BA 13) Right insula (BA 13)	−38 34	−2 12	8 10	5.83 5.43	248 166
Right amygdala (BA 24)	22	−2	−22	4.11	99
Right ACC	2	34	12	4.83	139

ACC, anterior cingulate cortex; BA, Brodmann area; HC, healthy control; IED, intermittent explosive disorder; MNI, Montreal Neurological Institute.

The regions exhibited the greater activity in the IED group compared to healthy controls during the anger-inducing condition compared to the neutral condition. There was no brain region in which healthy controls exhibited greater activation than the IED group.

MNI coordinates of the maximum t-scores are shown for each cluster.

Results are reported at p < 0.05, false discovery rate-corrected for the whole-brain analysis.

Multiple linear regression analysis was conducted to identify brain regions positively associated with the severity of aggression after adjusting for depression, anxiety, and impulsivity effects. The results were significant for the left anterior insula (BA 13) (*p* < 0.001, uncorrected) during anger-inducing conditions (**Table 5**). Post-hoc analyses using multiple linear regression revealed that only the composite aggression score was significantly positively correlated with the extracted percent signal change from the left insula during the anger-inducing condition, and the factors explained the variability of the left insula activation by 42.38%, as shown in **Figure 3B** and **Figure S1**.

**Table 5:** Functional magnetic resonance imaging (fMRI) result of whole-brain multiple regression analysis. The composite aggression value was positively correlated with the left insula.

Brain region	MNI coordinates			*t*_max_	Cluster size (voxels)
	x	y	z		
Left insula	−34	−2	4	4.88	35

MNI, Montreal Neurological Institute.

MNI coordinates of the maximum t-scores are shown for a cluster.

Results are reported at p < 0.001, uncorrected for the whole-brain analysis.

## Discussion

To the best of our knowledge, this is the first study to apply combined structural and functional MRI to identify altered brain regions in individuals with IED. We demonstrated that individuals with IED had significantly diminished gray matter volume in the anterior insula, amygdala, and orbitofrontal area, after statistically controlling for age and years of education, relative to controls. Gray matter volume in the left insula was negatively correlated with the severity of IED, as measured by the composite aggression score. We also examined altered brain function during anger processing among individuals with IED. fMRI revealed that patients with IED showed greater activation in the anterior insula, putamen, anterior cingulate cortex, and amygdala in response to anger-inducing films than did healthy controls. In addition, activation in the left insula during anger processing was associated with IED severity.

The regions we identified as exhibiting significantly greater activation in the IED group are known to be related to emotion processing functions such as emotional perception, experience, regulation, and reactions to anger (28, 29). The putamen, one of the structures comprising the basal ganglia, is linked to motor control, cognition, emotion, and somatosensory functions (30). According to previous neuroimaging studies on emotion, the putamen is associated with the recognition of emotional facial expressions (31–33). A growing body of evidence from animal studies (34, 35), lesion studies (36), and neuroimaging studies (37, 38) has suggested that the amygdala is associated with the subliminal perception of emotional stimuli, which includes the decoding of emotional reactions according to their significance. Several studies have implicated the anterior cingulate cortex in emotional regulation. According to a meta-analysis study, the anterior cingulate cortex is activated when detecting emotional conflicts and emotion appraisal (39). Moreover, the extent of reappraisal-mediated changes in the anterior cingulate cortex predicts the extent of negative affect attenuation (40). Therefore, the greater activation of these regions in the IED group than controls in the present cohort may suggest that anger arousal was stronger in the IED group.

Previous fMRI studies on pathological aggression have suggested a cortico-limbic model that implicates hyperreactivity in brain areas related to emotional expression (e.g., amygdala and insula) and hyporeactivity in brain regions involved in emotional regulation (e.g., orbitofrontal cortex and anterior cingulate cortex) (7, 41, 42). Our fMRI results partially support the cortico-limbic model of pathological aggression with respect to the hyperresponsivity of subcortical regions. We found hyperreactivity in brain regions involved in both, emotional expression, and regulation. The activation of certain brain areas in our fMRI study may be related to task-related behavioral aggression rather than pathological aggression. Since these types of aggression may be confounded, we cannot be certain whether the present fMRI results are restricted to the neural correlates of pathological aggression in IED. KrKr K et al. (2007) demonstrated that individuals who reported greater anger during the task showed greater provocation-related activity in brain regions that support emotional expression (insula) and increased activity in emotional regulation regions (i.e., inferior frontal gyrus, anterior cingulate cortex) (43). In the current study, the IED group reported experiencing anger that was more intense relative to that reported by controls in response to anger-inducing stimuli. Therefore, to identify the areas specifically linked to pathological aggression rather than task-related behavioral aggression among these activated brain regions, we also examined whether IED severity was correlated with activity in these clusters. The finding suggested that the insula was positively correlated with pathological aggression in IED. The results of our correlation analysis provide supportive evidence regarding the important role played by the insula in IED.

VBM analysis in our cohort revealed reduced gray matter volumes in the left insula, amygdala, and orbitofrontal area among individuals with IED compared with controls. Similar to the fMRI results, alterations in the insula were associated with the severity of IED, as measured by the composite aggression score. These VBM results correspond to the findings of a previous VBM study on IED (5). In agreement with our VBM results, the findings from recent studies have provided new information on the role of the insula in impulsive aggression (5, 44–46). Tiihonen et al. (2008) observed reductions of focal gray matter volumes in the right insula among violent offenders when compared to healthy individuals (44). A study on substance use disorders reported that individuals with substance use disorders who exhibit violent behavior display smaller gray matter volumes in the left insula than those who do not (45). Other studies in aggressive individuals reported reductions in gray matter volume in the anterior insula and amygdala (46).

In our combined study, the core overlap of neural substrates was located in the anterior insula, suggesting anterior insula involvement in IED. The insula has afferent and efferent connections with the orbitofrontal cortices, anterior cingulate, and amygdala (47). Craig (2009) suggested that the anterior insula is a crucial node in the human awareness network (i.e., awareness of bodily sensations, interoception, and awareness of affective feelings), the main role of which is the subjective regulation of psychological and physiological responses to cognitively challenging conditions (48). More recently, Dambacher et al. (2014) and Dambacher, et al. (2015) suggested strong and constant involvement of the anterior insula in motor impulsivity and reactive aggression (49, 50). They found an overlap of activity in the anterior insula between failed motor response inhibition and reactive aggression in healthy men using functional imaging. By providing an overarching role of the anterior insula across different modalities of self-control, they suggested that the insula functions in a comprehensive model of impulsivity as an integrator of cognitive, social, and emotional factors (49, 50). Based on our findings and the known roles of the insula, we speculate that alterations in the insula, such as a reduction in gray matter volume and increased activity during anger processing, may be associated with the observed anger regulation deficits in individuals with IED. In particular, we found left lateralization for IED in the insula. The correlation results showed that the left insula was structurally and functionally impaired. This finding agrees with that of a recent meta-analysis, which demonstrated hemispheric asymmetry in the functional activation of the insula during emotional processing (30). This study showed left-lateralized insula activation during emotional perception and experience. Another study on emotional intelligence reported that its score was significantly correlated with the activity of the left insula during social judgment of fearful faces. These studies suggest that the alterations of the left insula may lead to psychiatric disorders by affecting changes in social and emotional functioning (51). Thus, it is possible that the left lateralized alteration of the insula in the IED group may link with a lower ability to process oneonetion of the insula in the IED group may link with a lowesocially inappropriate behavior characteristics.

This study has several limitations. First, we conducted a cross-sectional study; therefore, we could not identify any causal relationship between IED and alterations in the insula. Our study design prevented the evaluation of association between alterations in the insula and the development of IED, and insular changes consequent to impulsive and frequent aggression. Longitudinal studies will be necessary to identify causal relationships between alterations in the insula and development of IED. Second, we used 7T MRI to acquire anatomical data with high spatial resolution and functional data with high signal to noise. However, 7T MRI has certain limitations related to susceptibility, signal dropout, and distortion. We used several methods to compensate for these limitations; however, that did not ensure full compensation. Third, a relatively small number of individuals participated in the study. The small sample size and low statistical power may have affected the reliability of the findings. For this study, the sample size was calculated by evaluating in the study. The small sample size and low statistical power may 1, as determined by a previous 7T fMRI study (26). With G Power of 3.1.9.2 (52), the sample size 14 participants in each group was calculated to be appropriate. To allow for a 10% dropout rate per group, we enrolled 30 patients in total. However, to increase external validity and provide insights into IED that are more generalizable, larger sample sizes should be used in future studies. A hypothesis-generating study is necessary to gain an understanding of the population and determine the optimal sample size (53). Finally, to identify functional connections and interactions between the insula and other regions in the resting state, resting-state fMRI will be needed. Task-based fMRI studies may provide information on specific functional disturbances, while resting-state fMRI studies may elucidate different and potentially broader alterations, such as distributed circuit abnormalities in individuals with neuropsychiatric illnesses (54). Therefore, to validate the cortico-limbic model proposed by previous research on IED, resting-state functional connectivity analysis will be required.

Despite these limitations, the strength of our study lies in the combined use of fMRI and VBM to examine patients with IED, as such studies on brain changes in these patients using both approaches are scarce. By conducting this combined functional and structural brain imaging study, we were able to demonstrate that impulsive and frequent aggression are linked to changes in brain structure and function, thereby clarifying aspects of the underlying neurobiology of IED.

In summary, the present structural and functional study revealed gray matter deficits and altered functional activity in the insula among individuals with IED relative to healthy controls. In particular, the diminished structure and increased functional activity in the insula in response to anger stimuli were significantly associated with the severity of IED. These findings provide new insights into the underlying neural mechanisms of IED and suggest that alterations in the insula may act as neuroimaging markers of IED.

## Data Availability Statement

The data are not publicly available due to their containing information that could compromise the privacy of research participants. Requests to access these datasets should be directed to the corresponding author.

## Ethics Statement

All participants provided their written informed consent to participate in this study after being thoroughly informed about the details of the experiment. The studies involving human participants were reviewed and approved by: The experimental procedures were approved by the Chungnam National University Institutional Review Board (approval number: 201803-SB-041-01; Daejeon, South Korea). 

## Author Contributions

J-WS made substantial contributions to conception and design, acquisition of the data, and analysis and interpretation of the data. J-WS and CC were involved in drafting the manuscript and revising it critically for important intellectual content. J-WS and CC agree to be accountable for all aspects of the work in ensuring that questions related to the accuracy or integrity of any part of the work are appropriately investigated and resolved. All authors gave final approval of the version to be published.

## Conflict of Interest

The authors declare that the research was conducted in the absence of any commercial or financial relationships that could be construed as a potential conflict of interest.
