# Supplementary material for: Gray Matter Deficits and Dysfunction in the Insula Among Individuals With Intermittent Explosive Disorder
Source: Front Psychiatry. 2020 May 20;11:439. doi: 10.3389/fpsyt.2020.00439 (PMC7251158; doi:10.3389/fpsyt.2020.00439)
Supplement: Supplementary file 1 [file DataSheet_1.docx]

# Supplementary Materials

# 1. Supplementary Tables

**Table S1. Detailed summary statistics of clinical characteristics separated by group**

| **Variables** | **Group** | **Min** | **1st Quarter** | **Median** | **Mean** | **3rd Quarter** | **Max** |
| --- | --- | --- | --- | --- | --- | --- | --- |
| **BDI** | IED | 0.00 | 3.50 | 9.00 | 9.80 | 16.50 | 19.00 |
|  | HC | 0.00 | 0.50 | 2.00 | 4.87 | 9.00 | 14.00 |
| **BAI** | IED | 1.00 | 4.00 | 8.00 | 8.40 | 12.00 | 18.00 |
|  | HC | 0.00 | 1.00 | 3.00 | 4.67 | 4.50 | 16.00 |
| **BIS-II** | IED | 45.00 | 54.00 | 60.00 | 60.07 | 67.00 | 72.00 |
|  | HC | 56.00 | 62.50 | 68.00 | 67.00 | 71.00 | 78.00 |
| **LHA** | IED | 4.00 | 9.50 | 12.00 | 11.67 | 14.50 | 18.00 |
|  | HC | 0.00 | 2.50 | 4.00 | 4.73 | 7.00 | 12.00 |
| **SA** | IED | 14.00 | 19.00 | 21.00 | 21.93 | 26.00 | 28.00 |
|  | HC | 10.00 | 14.00 | 14.00 | 15.13 | 17.00 | 21.00 |
| **TA** | IED | 17.00 | 20.00 | 21.00 | 23.00 | 26.50 | 32.00 |
|  | HC | 8.00 | 14.00 | 15.00 | 16.00 | 19.00 | 22.00 |
| **AQ** | IED | 60.00 | 67.50 | 69.00 | 73.07 | 83.00 | 82.00 |
|  | HC | 29.00 | 36.00 | 47.00 | 47.93 | 77.00 | 58.00 |

Abbreviations: AQ, Aggression Questionnaire; BAI, Beck Anxiety Inventory; BDI, Beck Depression Inventory; BIS, Barrett’s Impulsiveness Scale II; HC, healthy controls; IED, Intermittent Explosive Disorder; LHA, Life History of Aggression Questionnaire; SA, State Anger; TA, Trait Anger.

# 2. Supplementary Figure

**Fig S1. The relationship between the composite aggression value and the brain regions shown the differences of brain activity between groups.**

**
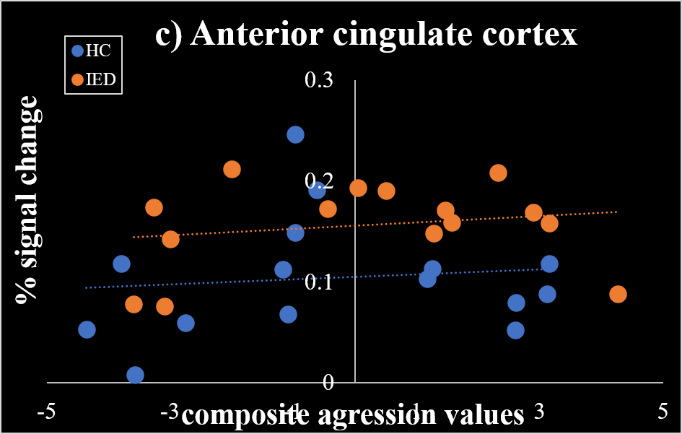

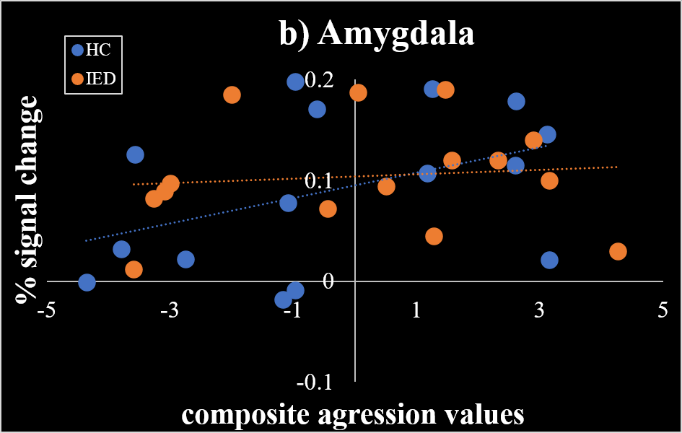

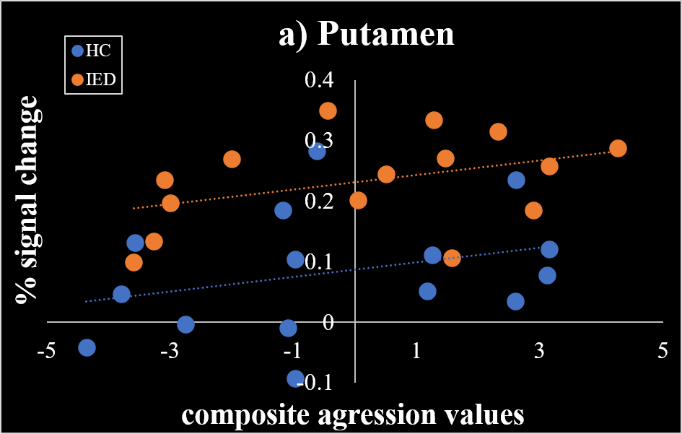
**

Scatter plots of the correlation between the composite aggression score and functional activity in the bilateral putamen, right amygdala, and right anterior cingulate cortex (*p* < 0.001, uncorrected). Each orange and blue circle represents the data of an IED and control participant, respectively.
